# Phenylbutazone concentrations in synovial fluid following administration via intravenous regional limb perfusion in the forelimbs of six adult horses

**DOI:** 10.3389/fvets.2023.1236976

**Published:** 2023-08-24

**Authors:** Molly O’Brien, Jonathan P. Mochel, Kevin Kersh, Chong Wang, Jarrod Troy

**Affiliations:** ^1^Department of Veterinary Clinical Sciences, Iowa State University College of Veterinary Medicine, Ames, IA, United States; ^2^Department of Biomedical Sciences, Iowa State University College of Veterinary Medicine, Ames, IA, United States; ^3^Department of Veterinary Diagnostic and Production Animal Medicine, Iowa State University College of Veterinary Medicine, Ames, IA, United States; ^4^College of Veterinary Medicine, University of Georgia, Athens, GA, United States; ^5^Department of Statistics, Iowa State University, Ames, IA, United States

**Keywords:** phenylbutazone, perfusion, horse, NSAID, anti-inflammatory, equine, IVRLP

## Abstract

**Background:**

Pain management is critical to equine welfare with non-steroidal anti-inflammatory drugs (NSAID) commonly used in horses. However, systemic NSAID use is limited by harmful gastrointestinal and renal side effects. Intravenous regional limb perfusion (IVRLP) is a technique used in horses that produces high, local antibiotic concentrations while limiting systemic circulation. NSAID-IVRLP would be a novel method of local pain management while limiting systemic NSAID side effects. To date, NSAID-IVRLP administration has not been reported in horses. This study aimed to identify the pharmacokinetics and local complications associated with using the NSAID phenylbutazone (PBZ) for IVRLP in six standing adult horses.

**Methods:**

PBZ-IVRLP, at a dose of 2.2 mg/kg PBZ, was performed in a randomly assigned forelimb cephalic vein in 6 standing, healthy adult horses. A placebo-IVRLP was performed in the contralateral forelimb for comparison. Systemic serum and radiocarpal joint synovial fluid PBZ concentrations were identified at various timepoints (before IVRLP T-0 h, just after tourniquet removal T-0.5, 1.5, 3, 5, 12, 16, and 24 h post IVRLP) for non-compartmental pharmacokinetic analysis and concentration over time curves. Local complications associated with PBZ-IVRP were evaluated for up to 7 days following PBZ-IVRLP using physical and ultrasonographic assessment. On day 7 horses were humanely euthanized with histology performed on both forelimbs at PBZ-IVRLP and placebo-IVRLP administration sites.

**Results:**

Non-compartmental pharmacokinetics for PBZ, and its major metabolite oxyphenbutazone (OBZ), were determined for serum and synovial fluid. Synovial PBZ concentrations (mean ± SD; 1.9 ± 2.1 μg/mL) were significantly lower (*p* = 0.03; CI,0.46–7.36) than serum PBZ concentrations (5.8 ± 5.1 μg/mL) at any time point. Physical and ultrasonographic measurements were not significantly different between PBZ- and placebo-IVRLP forelimbs. The most common histologic findings included focal deep dermal/subcutaneous hemorrhage and edema. Two horses showed perivasculitis and one horse showed a resolving thrombus in the cephalic vein of the PBZ-IVRLP limb. This horse also had severe perivasculitis and fibrinosuppurative dermatitis/panniculitis in the placebo-IVRLP limb.

**Conclusion:**

PBZ-IVRLP pharmacokinetics at a 2.2 mg/kg dose showed no benefit compared to systemic PBZ administration in standing adult horses. Local complications associated with PBZ-IVRLP were similar to placebo-IVRLP on physical and ultrasonographic evaluation.

## 1. Introduction

Phenylbutazone (PBZ), and other non-steroidal anti-inflammatory drugs (NSAIDs), are a major component of equine pain management and a mainstay for treating equine lameness (1–5). NSAIDs are effective, affordable, and easily administered, but carry significant health risks such as gastric ulceration, renal necrosis, and right dorsal ulcerative colitis (1–5). These side effects occur due to their non-selective, inhibition of cyclooxygenase (COX) pathways and severely restrict equine veterinarians’ ability to treat pain in the horse with few effective alternatives (1–7). Limiting these systemic side effects would greatly enhance the ability to treat equine pain and lameness.

Intravenous regional limb perfusion (IVRLP) is a common technique used in horses that generates high local drug concentrations in distal limbs while minimizing systemic concentrations (8, 9). Traditionally, IVRLP is used for antibiotic administration, but NSAID administration via IVRLP could allow for NSAID diffusion into local tissues while restricting systemic circulation and associated side effects (2, 5, 8, 9). However, NSAID-IVRLP has not been reported in horses nor is there a reported investigative model for any species. PBZ would be ideal for NSAID-IVRLP investigation as the pharmacokinetics (PK) for both PBZ and its major metabolite oxyphenbutazone (OBZ) are well reported in horses following systemic administration (2, 5, 10–12). OBZ is an analgesic metabolite of PBZ and occurs only after PBZ metabolism in the liver by cytochrome P450 (11, 12). Additionally, PBZ is anecdotally one of the most frequently used NSAIDs for equine pain management. Before PBZ-IVRLP clinical research and application can be performed it is important to identify PBZ-IVRLP pharmacokinetics and safety in the horse.

Anecdotally, safety concerns arise regarding NSAID-IVRLP due to potential risk of local tissue myositis/necrosis from extravascular PBZ concentrations. Tissue myositis/necrosis has been reported following extravascular PBZ injection in horses, but it is unknown if PBZ-IVRLP would result in similar complications (13). Traditional antibiotic IVRLP in the horse is relatively safe with minor complications, such as local site inflammation and/or thrombophlebitis, which have been evaluated using physical and ultrasonographic measurements (8, 9, 14). These measurements in conjunction with clinical signs and histopathology of the IVRLP site could be used to identify the local complications associated with NSAID-IVRLP (14).

The objectives of this study were to (1) determine the concentrations and PK of PBZ and OBZ in equine radiocarpal joint synovial fluid and systemic serum following PBZ-IVRLP administration, (2) identify local complications associated with PBZ-IVRLP using ultrasonography and clinical evaluation for 7 days following PBZ-IVRLP, and (3) identify histopathologic signs of necrosis, inflammation, and/or mineralization at IVRLP administration sites.

## 2. Materials and methods

### 2.1. Animals

All study procedures were approved by the Iowa State University Institutional Animal Care and Use Committee. Six healthy horses (three Quarter Horses, one Arabian, one Peruvian Paso, and one Paint) were used in this study and were donated for humane euthanasia for reasons unrelated to the aims of this study with expressed condition that they be euthanized at the conclusion of the study. Horses ranged from 1.5 to 28 years of age (mean, 17.4 years) with body weights ranging from 315 to 490 kg (mean, 420 kg). Horses were housed in stalls for the duration of the study. Inclusion criteria for the study protocol included no clinically important disease assessed by physical examination, obvious pathology in the forelimbs with musculoskeletal examination, and no observable lameness at the walk. Each horse had full range of motion for both carpi and no palpable evidence of radiocarpal joint osteoarthritis. No horse was administered oral or systemic intravenous NSAID or corticosteroids during the study. Horses only received PBZ administered via IVRLP.

### 2.2. Treatment protocol

Each forelimb was randomly assigned to receive only an IVRLP treatment with either placebo-IVRLP (saline solution [0.9% NaCl]) or PBZ-IVRLP, generating six limbs/treatment. Bilateral, IVRLP was performed once on each horse (14). Horses were sedated with xylazine (0.2–0.5 mg/kg, IV) and a median, ulnar, and medial cutaneous antebrachial nerve block was performed on each forelimb (15). Horses were then sedated with detomidine hydrochloride (0.01–0.02 mg/kg, IV) and butorphanol (0.01–0.015 mg/kg, IV). The cephalic vein of each forelimb was prepared with 4% chlorhexidine gluconate^a^ followed by 70% isopropyl alcohol.^b^ Both limbs were prepared in this manner prior to IVRLP. A 16 cm wide rubber tourniquet was applied 10 cm proximal to the accessory carpal bone. One roll of gauze was placed over the cephalic vein underneath the tourniquet. A 21 gage butterfly catheter^c^ was placed in the cephalic vein and the assigned perfusate administered over 3 min timed using a digital countdown timer. PBZ-IVRLP perfusate included 20% PBZ injectable solution^d^ (2.2 mg/kg dose) diluted to 60 mL with 0.9% NaCl. Placebo-IVRLP perfusate consisted of 60 mL 0.9% NaCl. A small pressure bandage was placed over both injection sites to prevent leakage from the blood vessel. Once the pressure bandage was secured the contralateral limb received the same tourniquet procedure and the assigned IVRLP. The first IVRLP treated forelimb was randomly assigned (i.e., either the PBZ-IVRLP or placebo-IVRLP may have been the first forelimb IVRLP of a horse) with ~1–2 min (timed with a hanging analog clock) between the first and contralateral limb IVRLP administration. Tourniquets were released 30 min after IVRLP administration and the pressure bandage was subsequently removed.

### 2.3. Synovial fluid and serum sample collection

Baseline radiocarpal synovial fluid (1–3 mL) was obtained using the dorsal approach from only PBZ-IVRLP treated limbs prior to IVRLP (T-0), 0.5 (just after tourniquet removal), 1.5, 3, 5, 12, 16, and 24 h post IVRLP. Sedation with xylazine (0.2–0.5 mg/kg, IV) was administered to facilitate sampling. Blood samples (10 mL) were collected from a jugular vein at the same time points as synovial fluid sampling. All blood samples were collected into anticoagulant-free tubes and centrifuged at 1,000 × *g* for 5 min. Serum supernatant and synovial fluid were transferred into cryogenic storage tubes and stored at −80°C.

### 2.4. Ultrasonographic measurement

A linear transducer (8–14 MHz) was used to image the IVRLP injection site and a 4 cm region proximal and distal to the injection site in transverse and longitudinal planes. Ultrasound scans were performed prior to IVRLP, immediately after IVRLP, and repeated every 24 h for 7 days on all limbs. Measurements included transverse and longitudinal imaging of cephalic vein diameter, cephalic vein wall thickness, and subcutaneous tissue thickness over the cephalic vein (13).

### 2.5. Clinical assessment and circumference measurement

Each horse was evaluated for clinical changes in demeanor, appetite, lameness, and signs of myositis or tissue necrosis during hospitalization. Criteria for clinical tissue myositis/necrosis included any one of the following conditions: grossly visible skin or subcutaneous tissue sloughing, exudate present at the IVRLP site, emphysema at the injection site more than 12 h after IVRLP, moderate–severe pain response to palpation more than 24 h post PBZ-IVRLP, or development of non-weight bearing lameness. A measuring tape was used on each forelimb at the level of the injection site before and after IVRLP to record circumference measurements by one of the investigators, who was unaware of limb treatment. These measurements were additionally recorded every 24 h for 7 days following IVRLP.

### 2.6. Phenylbutazone and oxyphenbutazone concentration analysis

Synovial fluid and serum samples were submitted to the Iowa State University Analytical Chemistry Services (ISU ACS) for analysis. Briefly, samples were processed by protein precipitation with acetonitrile. All samples were concentrated to dryness and reconstituted in a combination of organic solvent and water prior to injection on an analytical column. Separation was performed using reversed phase C18 analytical column. Analysis was performed on a LC–MS/MS system comprised of a triple quadrupole mass spectrometer. Quantitation of PBZ was performed using a stabile isotope labeled internal standard to produce a linear regression curve. Detailed method development/alterations may occur for concentration analysis between the upper and lower limits of quantification were performed following sample submission. Plasma upper limit of quantification (ULOQ) and lower limit of quantification (LLOQ) for PBZ and OBZ concentrations were set as follows: PBZ (ULQ = 100 ug/mL; LLOQ = 0.3 ug/mL) and OBZ (ULOQ = 15 ug/mL; LLQ = 0.3 ug/mL) (13, 15). Synovial fluid ULOQ and LLOQ for PBZ and OBZ concentrations was set as follows: PBZ (ULOQ = 10 ug/mL; LLOQ = 0.1 ug/mL) and OBZ (ULOQ = 2 ug/mL; LLOQ = 0.1 ug/mL) (15).

### 2.7. Histological examination

Horses were humanely euthanized on day 7 following ultrasonographic and physical measurement of their forelimbs. Following euthanasia, a 5 cm × 10 cm (width by length) section of tissue centered over the IVRLP administration site was excised which included skin, subcutaneous tissue, and the cephalic vein. The samples were placed in jars containing 10% formalin and submitted to the ISU Veterinary Pathology Comparative Pathology Core (CPC) Research Services for histological examination by a board certified veterinary pathologist unaware of limb treatment. Tissues for histopathology were placed in 10% neutral buffered formalin for 24 h and then transferred to 70% ethanol, trimmed, and processed routinely for H&E staining.

### 2.8. Pharmacokinetic analysis

Pharmacokinetic analysis of total PBZ and OBZ within serum and synovial fluid concentrations was performed using a statistical moment (i.e., non-compartmental) approach in commercial software (PKanalix, MonolixSuite 2020R1, Lixoft, France). Time versus concentration figures for PBZ and OBZ were produced via a commercial program (GraphPad Prism 8.0, Graphpad Software, Inc., La Jolla, CA, United States). Standard PK parameters were generated for individual horses as follows:

- Maximum PBZ and OBZ concentration, Cmax;
- Time of maximum PBZ and OBZ concentration, Tmax;
- Area under PBZ and OBZ concentration-time curve, AUC_last_;
- Area under the moment curve, AUMC_inf_;
- PBZ and OBZ mean residence time, MRT = AUMC_inf_/AUC_inf_;
- Slope of the elimination phase λz, computed by linear regression of the logarithmic concentration vs. time curve during the elimination phase, λ_z_;
- PBZ and OBZ terminal half-life, *T*_1/2_(λ_z_) = ln(2)/λ_z_;
- PBZ apparent clearance, CL/F = Dose/AUC_inf_;
- Apparent volume of distribution of PBZ during the elimination phase, *Vz/F* = Dose/(AUC_inf_ × λ_z_);

For data analysis, the first value below the LLOQ was inferred to be LLOQ/2, and subsequent data points were excluded from the analysis. A linear/log trapezoidal rule was used to estimate the area under PBZ and OBZ time curves. Summary statistics on the individual pharmacokinetic parameters were performed thereafter to derive the geometric mean, median, and (min-max) range.

### 2.9. Statistical analysis

Data distributions for all pharmacokinetic parameters, limb circumference, and ultrasonographic measurements were normality assessed by Shapiro–Wilk tests. Drug concentrations were compared at each time point using contrasts. Comparison of variables between PBZ-IVRLP and placebo-IVRLP limbs that were single observations were made using a paired *t*-test when data were normally distributed and with a Wilcoxon signed rank test when distributions were not normally distributed via a commercial program (GraphPad Prism 8, GraphPad Software, Inc., La Jolla, CA, United States). Statistical significance was *p* < 0.05.

## 3. Results

### 3.1. Pharmacokinetics

**Figure 1:**
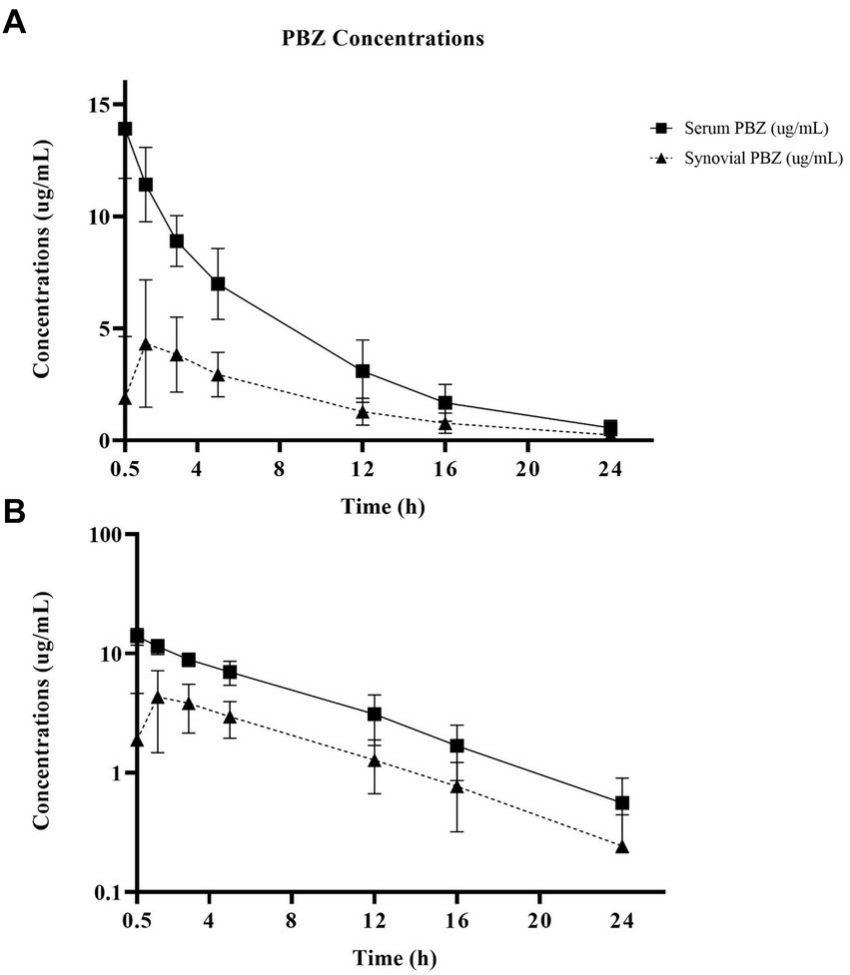
Linear **(A)** and semilogarithmic **(B)** plots of serum and radiocarpal joint synovial fluid concentrations of phenylbutazone (PBZ) over time following a single PBZ intravenous regional limb perfusion (IVRLP) using a 2.2 mg/kg PBZ dose in six adult equine forelimbs (mean ± SD). *X*-axis starts at tourniquet removal (T-0.5 h) sampling. No horse had PBZ concentrations at T-0 h.

**Figure 2:**
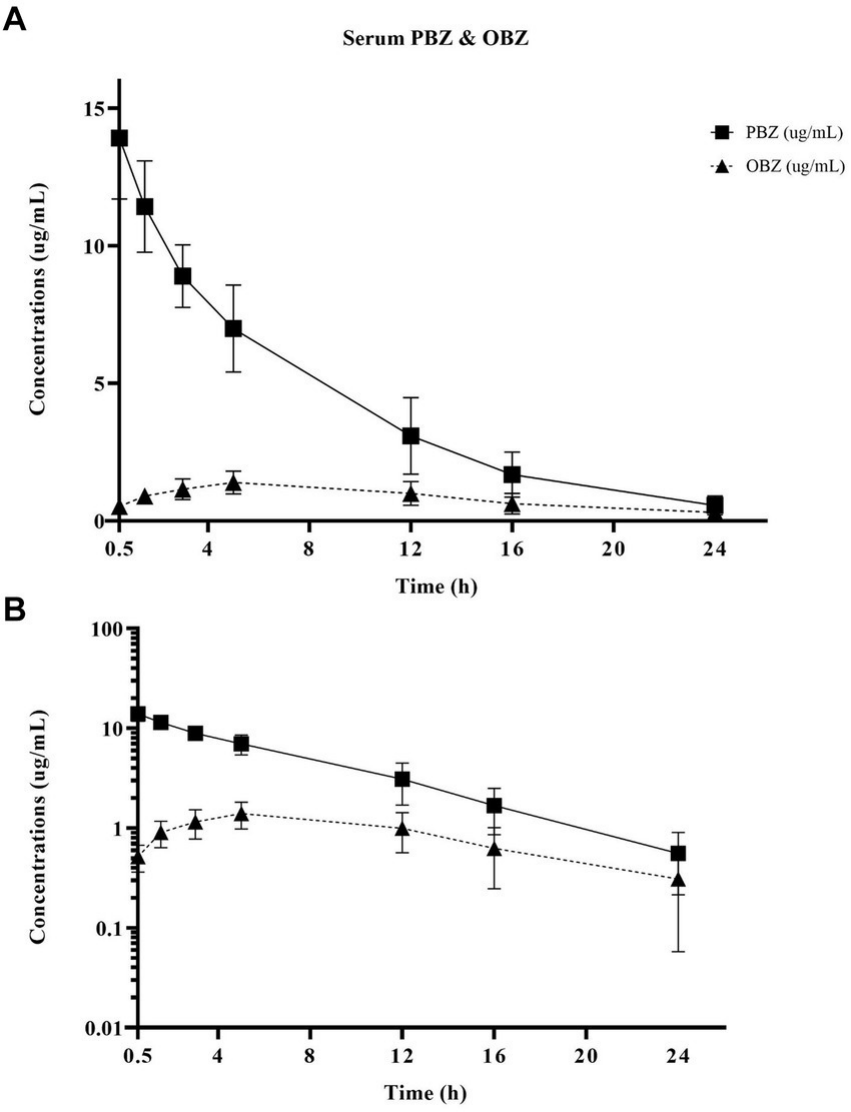
Linear **(A)** and semilogarithmic **(B)** plots of serum concentrations over time of PBZ and its metabolite OBZ after PBZ-IVRLP (2.2 mg/kg dose) in six adult equine forelimbs (mean ± SD).

**Figure 3:**
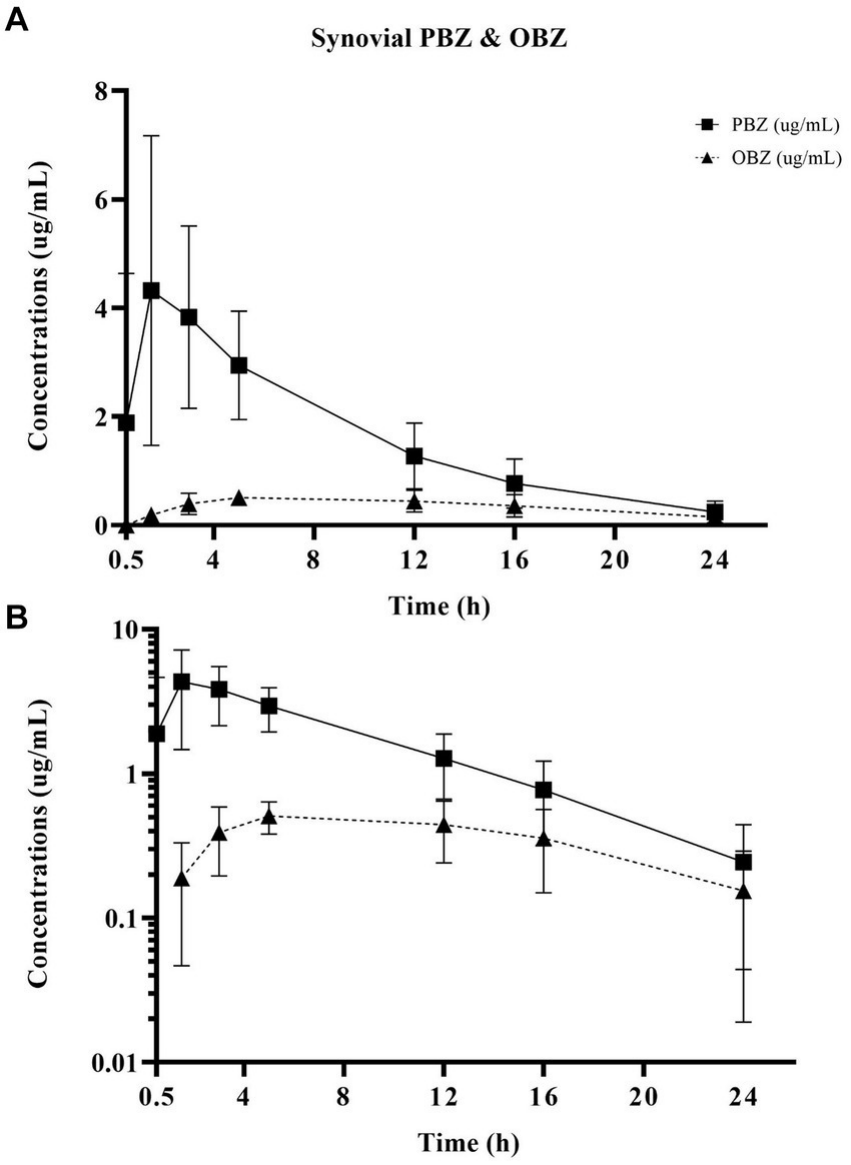
Linear **(A)** and semilogarithmic **(B)** plots of synovial concentrations over time of PBZ and its metabolite OBZ after PBZ-IVRLP (2.2 mg/kg dose) in six adult equine forelimbs (mean ± SD).

Non-compartmental PBZ pharmacokinetics for serum and synovial fluid after a single PBZ-IVRLP are summarized in Table 1. PBZ concentration over time curves are depicted in linear and semilogarithmic plots shown in Figure 1. Concentrations of PBZ and OBZ over time in relation to each other are shown in linear and semilogarithmic plots in both serum (Figure 2) and synovial fluid (Figure 3). No PBZ concentrations were detected in serum or synovial fluid before PBZ-IVRLP (0 h) in any horse. Maximum serum PBZ concentrations (Cmax, median [IQR], 14.8 μg/mL [12.7–15.4]) occurred at tourniquet removal (Tmax, 0.5 h). Time until synovial PBZ Cmax (3.9 μg/mL [3.3–5.4]) was longer (Tmax, 2.3 h [1.5–3 h]) than serum BPZ. Serum and synovial PBZ was still detectable at 24 h in 5/6 horses. In Horse 5, neither serum nor synovial PBZ was detectable at 24 h. Synovial PBZ concentrations (mean ± SD, 1.9 ± 2.1 μg/mL) were significantly lower (*p* = 0.03; CI, 0.46–7.36) than PBZ serum concentrations (5.8 ± 5.1 μg/mL). None of the horses had serum or synovial values for PBZ above the respective ULOQ throughout the study. For an LLOQ of 0.3 μg/mL, 2% (1/42) of the serum PBZ samples had values below the analytical quantification limit. At an LLOQ of 0.1 μg/mL, 2% (1/42) of the synovial PBZ samples were below the analytical quantification limit. Both serum and synovial samples below LLOQ were from the same horse at T-24 h.

**Table 1:** Serum and radiocarpal joint synovial fluid non-compartmental pharmacokinetic analysis parameters [median (interquartile range)] for phenylbutazone (PBZ) following a single PBZ intravenous regional limb perfusion (IVRLP)using a 2.2 mg/kg PBZ dose in six equine forelimbs.

Parameter	Serum PBZ	Synovial PBZ
AUC_inf_ (μg*h/mL)	106.2 [98–118.5]	43.6 [30.3–53.2]
AUC_last_ (μg*h/mL)	101.4 [95.3–113.5]	42.3 [28.9–47]
AUMC_inf_ (μg*h^2^/mL)	829 [689.3–984.8]	360.3 [249.7–376.3]
CL/F (mL*h^−1^/kg)	20.7 [18.6–22.5]	
Cmax (μg/mL)	14.8 [12.7–15.4]	3.9 [3.3–5.4]
Tmax (h)	0.5 [0.5–0.5]	2.3 [1.5–3]
*T*_1/2_ (h)	4.7 [4.5–5.4]	4.5 [4–5.7]
λ_z_ (h^−1^)	0.15 [0.13–0.15]	0.16 [0.12–0.18]
MRT_inf_ (h)	7.7 [7–8.5]	8.3 [6.4–12.2]
*V_z_/F* (mL/kg)	139.2 [132.4–170.7]	

All data was generated using non-compartmental analysis. AUC, area under the curve; AUMC, area under the moment curve; CL/F, clearance rate; Cmax, maximum concentration; Tmax, time until Cmax; *T*_1/2_, terminal half-life; λ_z_, terminal disposition rate constant; MRT, mean residence time; and *V_z_/F*, volume of distribution.

**Figure 4:**
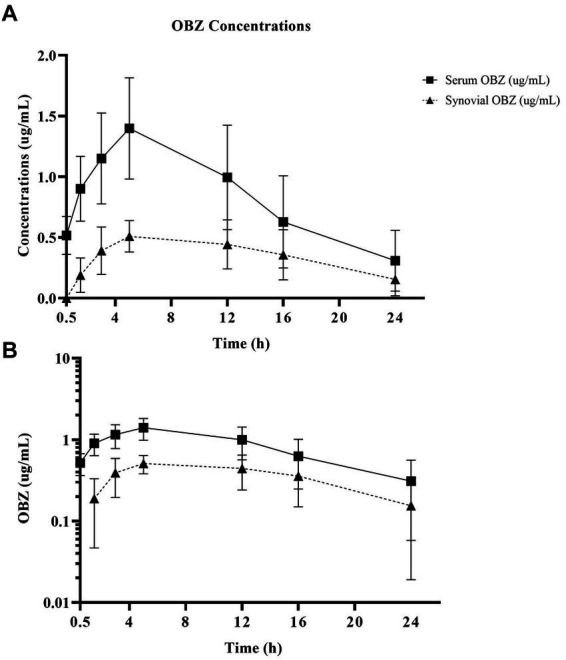
Linear **(A)** and semilogarithmic **(B)** plots of serum and radiocarpal joint synovial fluid concentrations of oxyphenbutazone (OBZ) after PBZ-IVRLP (2.2 mg/kg dose) in six adult equine forelimbs (mean ± SD).

Oxyphenbutazone pharmacokinetics for serum and synovial fluid are summarized in Table 2 with OBZ concentrations over time curves depicted in Figure 4. No OBZ concentrations were detected in serum or synovial fluid before PBZ-IVRLP in any horse. Synovial OBZ concentrations (0.26 ± 0.23 μg/mL) were significantly lower (*p* = 0.003; CI, 0.22–0.74) than serum OBZ concentrations (0.74 ± 0.52 μg/mL). The OBZ Cmax in serum (median [IQR],1.4 μg/mL [1.3–1.6]) was higher than synovial fluid (0.6 μg/mL [0.4–0.6]) but the Tmax for both serum and synovial OBZ was the same (5 h) (5). None of the horses had serum or synovial values for OBZ above the respective ULOQ throughout the study. For an LLOQ of 0.3 μg/mL, 7% (3/42) of the OBZ serum samples had values below the analytical quantification limit. For OBZ synovial samples, at an LLOQ of 0.1 μg/mL, 21% (9/42) had values below the analytical quantification limit.

**Table 2:** Serum and radiocarpal joint synovial fluid non-compartmental pharmacokinetic analysis parameters [median (interquartile range)] for oxyphenbutazone (OBZ) following a single PBZ intravenous regional limb perfusion (IVRLP) using a 2.2 mg/kg PBZ dose in six equine forelimbs.

Parameter	Serum OBZ	Synovial OBZ
AUC_inf_ (μg*h/mL)	24.4 [17.3–30.4]	10.4 [7.9–15.7]
AUC_last_ (μg*h/mL)	18.6 [12.5–23.8]	7.6 [5.3–10.9]
AUMC_inf_ (μg*h^2^/mL)	429.2 [220.5–511.5]	203.7 [115.5–235.2]
Cmax (μg/mL)	1.4 [1.3–1.6]	0.6 [0.37–0.63]
Tmax (h)	5 [5]	5 [5]
*T*_1/2_ (h)	8.3 [7.7–9.2]	9.2 [6.9–12.6]
λ_z_ (h^−1^)	0.09 [0.08–0.09]	0.08 [0.06–0.1]
MRT_inf_ (h)	14.8 [12.8–16.8]	17 [13.4–22.1]

All data was generated using non-compartmental analysis. AUC, area under the curve; AUMC, area under the moment curve; CL/F, clearance rate; Cmax, maximum concentration; Tmax, time until Cmax; *T*_1/2_, terminal half-life; λ_z_, terminal disposition rate constant; MRT, mean residence time; and *V_z_/F*, volume of distribution.

### 3.2. Physical evaluation and ultrasonography

Physical examinations were unremarkable in all horses throughout the study. None of the horses showed clinical signs of tissue myositis/necrosis or observable lameness at the walk throughout the study in either forelimb. During IVRLP, Horse 1 fell to its carpi but quickly regained his stance. The time when this occurred was not recorded and the horse stood quietly for the remainder of the IVRLP. Additionally, Horse 3 and Horse 4, displayed excessive movement during IVRLP, respectively, and continued to ambulate despite repeated doses of IV sedation.

Circumference measurements were similar (*p* = 0.48; CI, −0.45-0.92) in treated (30 ± 1.7 cm) and placebo (29 ± 1.6 cm) limbs throughout the study. There was no significant difference in ultrasonographic measurements of cephalic vein diameter, cephalic vein wall thickness, or subcutaneous tissue thickness between treated or placebo limbs. There was no difference in cephalic vein or subcutaneous tissue measurements before or after IVRLP in PBZ-IVRLP limbs. However, in placebo-IVRLP limb longitudinal scans, mean cephalic vein wall thickness was significantly greater (*p* = 0.04; CI, 5e^−4^-0.03) after IVRLP (0.07 ± 0.01 cm) than before IVRLP (0.06 ± 0.01 cm).

### 3.3. Histology

**Figure 5:**
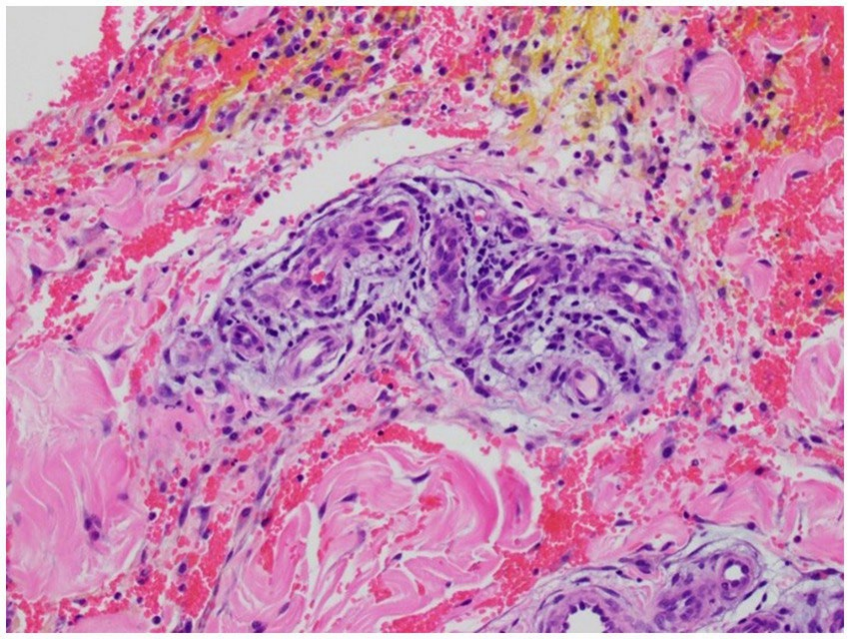
Histologic image of Horse 4’s phenylbutazone-IVRLP treated limb showing lymphoplasmacytic perivasculitis with dermal hemorrhage and edema (200X magnification).

**Figure 6:**
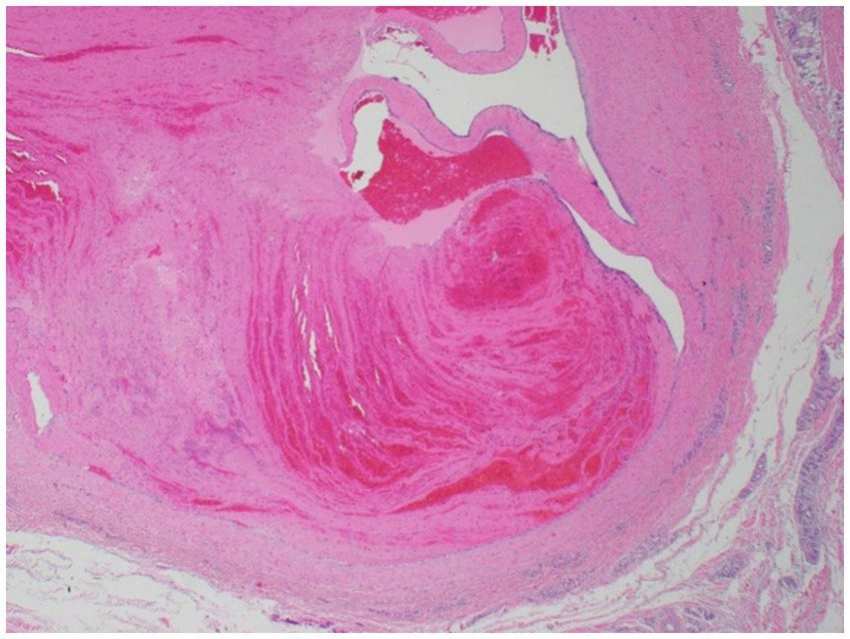
Histologic image of Horse 6’s phenylbutazone-IVRLP treated limb showing cephalic vein thrombosis with partial recanalization (40X magnification).

The most common histologic findings included focal deep dermal/subcutaneous hemorrhage (two placebo; two treated), edema within dermal, subcutaneous, and/or perivascular tissues (one placebo) or a combination of hemorrhage and edema occurring in five horses (three placebo; two treated). Perivasculitis was observed in two horses, Horse 4 and Horse 6. Horse 4 showed lymphoplasmacytic perivasculitis with dermal hemorrhage and edema in the treated limb (Figure 5) while a suppurative perivasculitis with dermal hemorrhage was identified in the placebo limb. Horse 6 demonstrated leukoclastic perivasculitis and fibrinosuppurative dermatitis/panniculitis in conjunction with deep dermal hemorrhage, edema, and lymphangiectasia in the placebo limb whereas the treated limb revealed cephalic vein thrombosis with partial recanalization (Figure 6).

The histologic findings between placebo and treated limbs were similar in 4/6 horses but disparate findings were identified in Horse 5 and Horse 6. Horse 5’s placebo limb showed deep dermal hemorrhage while its treated limb had no significant histologic changes identified. Horse 6 histologic limb disparities are described in the paragraph above.

## 4. Discussion

The primary objective of this paper was to determine the concentrations and pharmacokinetics of PBZ, and its active metabolite OBZ, in equine serum and synovial fluid following a single administration PBZ-IVRLP administration in standing adult horses. The administration of PBZ-IVRLP in standing adult horses resulted in serum PBZ concentrations that were significantly higher than in RCJ synovial fluid in this study. This is in contrast to reported synovial and serum antibiotic concentrations following antibiotic-IVRLP administration in horses (8, 9, 16). In this study, serum PBZ clearance, terminal half-life, and volume of distribution (CL/F, *T*_1/2_, *V_z_/F*) were similar to those reported following systemic PBZ administration (11, 12). The Tmax and Cmax for serum PBZ may have been similar to systemic administration as well but no serum samples were taken during the IVRLP procedure which is a limitation of this study. These findings show that PBZ remained within the peripheral vasculature more than it diffused into the synovial fluid of the RCJ. The *T*_1/2_ for synovial PBZ concentrations was similar to systemic serum indicating similar release of PBZ from the RCJ into systemic serum. This would indicate that the dosing schedule would likely need twice a day administration which may not be clinically practical or that PBZ is not tissue soluble enough to diffuse into the RCJ within the short tourniquet time of 30 min (11, 12). Additionally, OBZ concentrations were similar to those previously reported following systemic PBZ administration (12). These findings demonstrate that a single PBZ-IVRLP, at 2.2 mg/kg), was similar to a single systemic intravenous administration of PBZ in healthy, standing adult horses with no additional clinical benefits.

Although synovial PBZ concentrations were significantly lower than serum concentrations, it is unknown if these synovial PBZ concentrations would provide effective pain management. While the analgesic serum concentration of PBZ in horses is reported the analgesic synovial concentration is unreported (11). Indeed literature on synovial PBZ is sparse with only a single report describing synovial PBZ concentrations following oral PBZ administration consecutively for 2 weeks with synovial samples only taken at 0, 14, and 30 days, which makes direct comparison challenging (17). However, synovial PBZ concentrations (1.9 ± 2.1 μg/mL) from this current study were somewhat higher than those reported following 14 consecutive days of oral PBZ administration (0.93 ± 0.13 μg/mL) (17). A future study identifying synovial PBZ concentrations after oral or IV administration at 2.2 mg/kg would be helpful in determining if PBZ-IVRLP does produce higher synovial concentrations than systemic administration (17, 18). A limitation of this study is that there was no control group of horses that received systemic PBZ for comparison with IVRLP administration. The dose of 2.2 mg/kg PBZ was chosen for this study as that is the current low dose of the reported 2.2–4.4 mg/kg clinical dose range in horses and could be used as a dose as other studies have found efficacy when using this dose systemically (12, 18). Using information from this study in correlation with the results of synovial PBZ concentrations after systemic administration at clinically effective doses (i.e., 2.2 mg/kg) would allow the use of PK/PD modeling to identify if PBZ-IVRLP at a dose lower than 2.2 mg/kg would provide analgesic synovial concentrations which would reduce the overall systemic PBZ amount (18, 19). Even with changes in PBZ-IVRLP may not address that PBZ does not diffuse out of peripheral circulation into the RCJ within the 30 min of tourniquet time and thus having no clinical difference than systemic PBZ administration.

This study elected to evaluate synovial concentrations for PBZ concentrations as that is what has been reported for antibiotic-IVRLP and used in NSAID evaluation studies of canine osteoarthritis (15, 20–27). This synovial sampling does not however take into account inflammation in the periarticular or perivascular tissues, which can be a component of arthritic pain, which is a limitation of this study. Future studies could assess inflammatory biomarkers in these tissues or measure tissue sample PBZ concentrations in the treatment area to identify PBZ-IVRLP effects on these regions. Additionally, PBZ-IVRLP, even if clinically applicable, would not address systemic inflammation and any effects it may have on limb pain in horses.

Forelimb movement may have affected the synovial PBZ concentrations in this study. The effectiveness of the tourniquet in isolating target vasculature is directly related to subtourniquet pressures, which can decrease due to limb movement during IVRLP (9, 20). Limb movement during IVRLP is a limitation of this study and a potential reason for the PBZ synovial concentrations as 3/6 horses in this study were recorded with limb movement during the IVRLP. The movement was recorded as excessive in two horses with one horse recorded as falling to its carpi. The number of movements was not recorded, which is a limitation of this study and should be included in future investigations. While three horses displayed excessive movement during the PBZ-IVRLP procedure, the remaining horses stood quietly during the IVRLP and had similar PBZ concentrations as those that moved. Perineural anesthesia was performed to reduce limb movement but has not been reported for bilateral IVRLP (21). It may be the lack of sensation of both forelimbs that led to this movement. Alternatively, it may be the bilateral tourniquet placement that caused limb movement as tourniquets can lead to limb numbness or a tingling sensation in humans (22). However, one report describes the use of bilateral tourniquets for IVRLP for the evaluation of local IVRLP complications but does not report horse/limb movement or that bilateral perineural anesthesia was performed (14). Bilateral IVRLP was performed in this study so that ultrasonographic measurements and tissue collection for histology could occur on both limbs at the same time for complication evaluation on similar timelines. Future PBZ-IVRLP studies could perform unilateral IVRLP or when involving bilateral IVRLP could combine local anesthetic with IVRLP perfusates to observe if there is a difference in horse movement (21, 23).

Variables with the IVRLP technique may also have impacted the synovial PBZ concentrations from this study as the optimal IVRLP technique is unknown (9, 16, 20, 24, 25). In this study, a wide-rubber tourniquet was chosen to mimic both field and hospital settings for equine veterinarians. Wide rubber tourniquets have been shown to produce adequate subtourniquet pressures and antibiotic synovial concentrations, however, a pneumatic tourniquet could have been used instead of a wide-rubber tourniquet (20, 25). However, pneumatic tourniquets may not be feasible in field settings and are relatively expensive when compared to wide rubber tourniquets. The application of a distal tourniquet may have also influenced RCJ PBZ concentrations (25). Schoonover et al. (25) found that placement of a second tourniquet just below the carpus produced significantly higher RCJ concentrations. However, Bergstrom et al. (26) recently reported that amikacin concentrations were higher in RCJ synovial fluid when using a single proximal tourniquet versus a proximal and distal tourniquet. The evaluation of tourniquet application or sampling of multiple synovial structures would be beneficial in future studies to identify the impact on PBZ concentrations.

Another cause of the resulting synovial PBZ concentrations may be PBZ’s inherent drug properties. There are numerous reports regarding antibiotic-IVRLP in horses but there is scant literature reporting the impact of the administered drug properties effects on IVRLP. Traditionally, antibiotic-IVRLP (often aminoglycosides) in horses produces locally high antibiotic concentrations while minimizing systemic circulation (9, 16, 24, 25). However, data reported here shows this did not occur following PBZ-IVRLP. This may be due to the difference in protein binding between PBZ (98% protein binding) and aminoglycosides (0–30%) (28, 29). Non-protein bound, or “free,” drug form can cross the endothelium through simple diffusion whereas the protein bound form is restricted by healthy endothelial tight junctions (27–30). This changes in the presence of inflammation when tight junctions weaken allowing protein extravasation (31). This has been reported for PBZ in inflammatory exudate and for the highly protein bound NSAID robenacoxib in dogs (27, 28, 30). Silber et al. (27) found that robenacoxib concentrations were significantly higher in canine synovial fluid of inflamed joints vs. non-inflamed joints in dogs administered systemic robenacoxib. It may be that PBZ-IVRLP did not produce significantly higher synovial PBZ concentrations in the population of the horses studied here lacked systemic or local inflammation to weaken tight junctions. These horses had no clinical signs of systemic inflammation, no fevers, no lameness at the walk, no RCJ effusion, and full range of motion which would indicate clinically that RCJ inflammation was unlikely. However, no carpal radiographs, synovial fluid analysis, or systemic bloodwork were performed to confirm the lack of local or systemic inflammation which is a limitation of this study. Determining PBZ concentrations following IVRLP in horses with concurrent systemic or synovial inflammation would greatly aid in identifying the efficacy of PBZ-IVRLP in horses.

A secondary objective was to compare local complications between PBZ-IVRLP and a placebo-IVRLP administration assessed with physical examination, ultrasonography, and histology. In this study, local complications between PBZ-IVRLP and placebo-IVRLP were similar when assessed using ultrasonography, physical examination, and histopathology. Local antibiotic-IVRLP complications include local inflammation, target vein phlebitis, thrombosis, or hematoma, and have been previously investigated using physical and ultrasonographic evaluation (8, 14). The results from this study showed that physical and ultrasonographic findings were similar between PBZ-IVRLP and placebo IVRLP limbs for up to 7 days post-IVRLP. Ultrasonographic measurements of antibiotic-IVRLP in horses have been reported which showed a difference in subcutaneous tissue thickness (14). However, that study was evaluating the effect of a 1% liposomal diclofenac cream following antibiotic-IVRLP which may have reduced subcutaneous tissue thickness in treatment limbs compared to controls (14). It may be that 1% diclofenac cream will also reduce subcutaneous tissue thickness following PBZ-IVRLP but was not performed to remove any risk of the two NSAIDs interfering with concentration analysis. Subjective regional inflammation scoring was also performed in that study but was not performed in this study (14). Interestingly, while there was no difference between the PBZ-IVRLP and placebo-IVRLP; there was a difference in cephalic vein wall ultrasound thickness within the placebo-IVRLP treatment group. In the placebo-IVRLP group, post-IVRLP cephalic vein wall thickness was greater than pre-IVRLP thickness. The reason for this difference is unclear and may be related to the ultrasound equipment utilized or challenges in operator measurement as this was the smallest area measured in the study. Adaptations to the cephalic vein wall measurement could include screen enlargement or zoom capabilities on the cephalic vein wall. Additionally, a standoff pad was not used for ultrasonographic measurements, which is a limitation of this study, as both the skin and target structures deep to the skin appeared to be readily identifiable on ultrasound.

Histologic findings overall between groups was similar between limbs with most horses (4/6) showing minor complications associated with hemorrhage or edema except for Horse 4 and Horse 6. Horse 6’s findings are described above and Horse 4 showed perivasculitis at the administration site in both limbs. This horse had normal cephalic vein blood flow and no clinical signs of tissue necrosis or lameness. These findings were similar between PBZ-IVRLP and placebo limbs except for Horse 5 and Horse 6. Horse 5 had dermal hemorrhage in the placebo limb whereas the PBZ-IVRLP had no significant histologic findings. There are no histologic reports on the local complications following antibiotic-IVRLP in horses so direct comparison to results from this study is not possible. The results of this study may indicate that PBZ-IVRLP is similar to placebo-IVRLP or possibly antibiotic-IVRLP in healthy standing horses. However, future studies should still involve evaluation of regional complications as the PBZ concentrations in this study show that most of the PBZ remained intravascular following PBZ-IVRLP. It may be that if more PBZ extravasated it may cause local necrosis/myositis. A limitation of this study is that the limbs studied were treated with either PBZ or placebo IVRLP with no untreated control limb for evaluation. Additionally, the true incidence of complications of perivascular PBZ administration is unknown and although these six horses did not show complications associated with perivascular PBZ that may not hold true for a larger number of horses. Continued evaluation of perivascular PBZ complications should continue in any future PBZ-IVRLP studies.

Limitations of this study include the small number of horses used and the presumption that these horses were healthy without RCJ or systemic inflammation. However, no systemic bloodwork, inflammatory biomarker assessment, or diagnostic imaging was performed to confirm these presumptions. Future studies could include the use of systemic bloodwork and radiography of the studied joints to evaluate for underlying sub-clinical disease. Pursuing systemic bloodwork in future studies would also aid in identifying if there are systemic changes regarding PBZ administration (e.g., renal values, albumin, and total protein), which were not evaluated in this study (2–5). Also this study did not evaluate synovial PBZ’s effect on cartilage as it was outside the scope of this study and would be challenging to identify pre-IVRLP without inducing synovial inflammation. Beluche et al. (17) found that PBZ can decrease cartilage proteoglycan synthesis and future studies evaluating this effect following PBZ-IVRLP would be useful. This study also acknowledges the limitation of not identifying the free drug concentrations of PBZ, which is highly protein bound, in serum or synovial fluid of horses administered PBZ-IVRLP. This could be incorporated in future studies. In this study a dose of 2.2 mg/kg was chosen because it is the lowest reported clinical dose for efficacy and was the only dose used in this study which is a limitation of this study (18). Future studies could include alternative dosing of PBZ clinical doses or dose scaling for the tissue volume of metabolic tissue being perfused. Additionally, this was a single dose administration to identify the pharmacokinetics following a single dose which is not clinically relevant by itself, and the results here indicate that PBZ-IVRLP was similar to systemic administration. Another limitation of this study was that no synovial nor systemic serum samples were taken during the IVRLP procedure to identify if the tourniquet was effective in isolating the target vasculature. Future studies should include sampling times of both synovial fluid and systemic serum during the IVRLP procedure itself. This study also acknowledges that a butterfly catheter was used for the IVRLP instead of an over the needle catheter. This was chosen as the authors are more familiar with the use of the butterfly catheter for IVRLP and also as a butterfly catheter was chosen for another study comparing local IVRLP complications with ultrasonography (14). Future studies could compare the difference between butterfly catheter and over the needle administration of IVRLP in horses. The authors also acknowledge that this study evaluated a single administration of PBZ-IVRLP and that there was no assessment for repeated administration which is common in horses with orthopedic pain. It may be that repeated PBZ-IVRLP administration would have resulted in local complications not seen in this study. Additionally, from the T_1/2_ of synovial PBZ determined in this study the dosing regimen for PBZ-IVRLP would be more frequent than every 24 h which calls into practicality of this technique in a clinical setting. The authors further acknowledge that this study lacked a control population of horse forelimbs that did not receive either PBZ-IVRLP or placebo-IVRLP to be assessed via ultrasonography or histology. In this study, bilateral IVRLP was performed which has only been reported by Levine et al. (14) instead of performing IVRLP on a single forelimb which is one of this study’s limitations. Lacking a control group of adult horses administered systemic PBZ for comparison was an additional limitation to this study and acknowledged by the authors.

Overall, these findings report the pharmacokinetics of PBZ and OBZ after PBZ-IVRLP, at a 2.2 mg/kg dose, in standing healthy adult horses which demonstrate no clinical advantage to intravenous systemic PBZ administration when using the described IVRLP technique. Although PBZ-IVRLP showed no significant difference in local complications compared to placebo-IVRLP in this study that may be due to the majority of PBZ remaining within peripheral vasculature during IVRLP. Future studies would be needed to further assess local complications with PBZ-IVRLP.

### 4.1. Products and equipment details

- ChlorHex Maxi Scrub 4%, VEDCO, Inc., St. Joseph, MO, United States
- Isopropyl alcohol 70%, Clipper Distributing Company, LLC., St. Joseph, MO, United States
- Surflo Winged Infusion Set, Terumo Corporation, Tokyo, Japan
- Phenylbutazone 20%, MWI Animal Health, Boise, ID, United States

## Data availability statement

The raw data supporting the conclusions of this article will be made available by the authors, without undue reservation.

## Ethics statement

The animal study was approved by Iowa State University Internal Animal Care and Use Committee. The study was conducted in accordance with the local legislation and institutional requirements.

## Author contributions

MO’B and KK contributed to animal acquisition, data collection, interpretation of all information, and revised multiple drafts with conceptualization. JT contributed to work conception/design, animal acquisition, data collection, interpretation of all information, pharmacokinetic analysis, and revised multiple drafts with conceptualization. JM contributed to pharmacokinetic analysis, interpretation of pharmacokinetic/pharmacologic results, and revised multiple drafts with conceptualization. CW contributed to statistical analysis of the data, statistical interpretation, and revision of multiple drafts. All authors contributed to the article and approved the submitted version.

## Funding

This work was supported by the USDA National Institute of Food and Agriculture, USDA Capacity: Animal Health and Disease Research Program, grant number AWD-025124-00001.

## Conflict of interest

The authors declare that the research was conducted in the absence of any commercial or financial relationships that could be construed as a potential conflict of interest.

## Correction note

A correction has been made to this article. Details can be found at: 10.3389/fvets.2026.1869864.

## Publisher’s note

All claims expressed in this article are solely those of the authors and do not necessarily represent those of their affiliated organizations, or those of the publisher, the editors and the reviewers. Any product that may be evaluated in this article, or claim that may be made by its manufacturer, is not guaranteed or endorsed by the publisher.
